# Correlation of asprosin and Nrg-4 with type 2 diabetes Mellitus Complicated with Coronary Heart Disease and the Diagnostic Value

**DOI:** 10.1186/s12902-023-01311-8

**Published:** 2023-03-13

**Authors:** Min Zhong, Xue Tian, Qitian Sun, Lihui Li, Yanan Lu, Zengbin Feng, Yu Gao, Shuying Li

**Affiliations:** 1 Department of Endocrinology, Affiliated Hospital of Chengde Medical University, Chengde, China; 2 Department of Cardiac surgery, Affiliated Hospital of Chengde Medical University, Chengde, China; 3 Department of Nursing , Affiliated Hospital of Chengde Medical University, Chengde, China

**Keywords:** Type 2 diabetes mellitus, Coronary heart disease, Asprosin, Neuregulin-4

## Abstract

**Purpose:**

Asprosin is a newly discovered adipose factor secreted by white fat, which is involved in glucose metabolism and inflammation. Neuregulin-4 (Nrg-4) is a new adipose factor released from brown adipose tissue and is considered to play an important role in metabolism. This study aims to explore the association between serum Asprosin, Nrg-4 level and coronary heart disease(CHD) in patients with type 2 diabetes mellitus(T2DM) and the diagnostic value.

**Patients and methods:**

157 patients with T2DM were enrolled from Affiliated Hospital of Chengde Medical University between December 2020 to July 2021. These patients were divided into T2DM without CHD group (T2DM-0, n = 80) and T2DM with CHD (T2DM-CHD, n = 77). Serum Asprosin and Nrg-4 expression was detected by enzyme-linked immunosorbent assay, and the correlations between Asprosin or Nrg-4 and clinical and biochemical indicators were analyzed. A receiver operating characteristics curve analysis and area under the curve (AUC) were used to evaluate diagnostic accuracy.

**Results:**

Serum Asprosin level of the T2DM-CHD group were significantly higher and Nrg-4 level significantly lower than those of the T2DM-0 group.Spearman correlation analysis showed that serum Asprosin levels were significantly positively correlated with diabetes course,history of hypertension, fasting plasma glucose(FPG), glycosylated hemoglobin A1c(HbA1C), triglycerides(TG),triglyceride glucose index(TyG index) and urea, and negatively correlated with ALT (all *p* < 0.05). Nrg-4 was negatively correlated with history of hypertension, body mass index(BMI), FPG, HbA1C, TG, and TyG indexes (all *p* < 0.05), and positively correlated with high-density lipoprotein cholesterol(HDL-C)(*p* < 0.05).Logistic regression analysis showed that after adjusting potential confounders, Asprosin was a risk factor for diabetes mellitus, Nrg-4 was a protective factor.The AUC of Asprosin for diagnosing T2DM-CHD was 0.671 (95% confidence interval [CI] 0.584–0.759), and the AUC of the Nrg4 index for diagnosing T2DM-CHD was 0.772 (95% CI 0.700-0.844). The AUC of Asprosin and Nrg-4 for the combined diagnosis of T2DM-CHD was 0.796 (95% CI 0.726–0.864).

**Conclusion:**

Asprosin and Nrg-4 may be novel diagnostic biomarkers for T2DM with CHD, as they effectively improved the diagnostic accuracy for T2DM-CHD.

## Introduction

Diabetes mellitus(DM) is a chronic non-communicable disease that threatens human health all over the world. About 1.164 billion Chinese suffer from DM, a number that is projected to increase to 1.405 billion in 2030. Over 90% of these patients suffer from type 2 diabetes mellitus(T2DM). A number of diabetic complications, particularly those related to the cardiovascular system, have captured the attention of researchers due to the rising incidence rate of T2DM[1]. A major risk factor for T2DM morbidity and mortality is coronary heart disease(CHD), which is the most common cardiovascular complication of T2DM [2]. As a consequence, identifying T2DM-CHD novel risk markers may prove beneficial for the effective treatment and prevention of cardiovascular complications associated with T2DM.

Asprosin is a 140-amino-acid C-terminal profibrillin, which is predominantly secreted and expressed by white adipose tissue. It has been shown that Asprosin could be induced by fasting and recruited to the liver, resulting in rapid liver glucose release into the circulation [3]. Researchers found that elevated asprosin levels were observed in people and mice with insulin resistance, T2DM, or obesity [4–6]. Furthermore, asprosin levels are pathologically elevated in cardiovascular disease (CAD) patients, correlated with adiposity, dyslipidemia, and insulin resistance, suggesting a possible link between asprosin and CAD pathophysiology [7]. Asprosin appears to be a promising therapeutic target for metabolic disorders based on the current research. The level of aspartate aminotransferase in patients with T2DM-CHD, however, has not been studied.

Neuregulin 4 (Nrg-4), an adipokine secreted by the brown adipose tissue (BAT) [8]. It belongs to the group of extracellular ligands known as epidermal growth factors (EGFs), which regulate cell-cell interactions within the nervous system, heart, chest, and other organ systems [9]. The biological functions of Nrg-4 include the inhibition of apoptosis, the promotion of neurite outgrowth, and the inhibition of inflammation [10, 11].

Researchers have reported an association between decreased Nrg-4 levels and T2DM mellitus, obesity, insulin resistance (IR) and hyperglycemia [12, 13]. Nrg-4 has been demonstrated to have anti-atherogenic and anti-inflammatory properties in recent studies [14]. As a result, it may provide evidence that diabetes and cardiovascular risk are related.

However, to date, there is no data on the role of Asprosin and Nrg-4 in diabetes and CHD. In this study, we explored the changes in Asprosin and Nrg-4 levels in patients who have either T2DM alone or CHD secondary to T2DM in order to develop effective diagnostic and predictive strategies. Furthermore, the correlation between Asprosin and Nrg-4 was evaluated in relation to other routine biochemical parameters. The results of this study may provide new ideas and methods for the prevention of T2DM complicated with CHD.

## Materials and methods

### Study population

In this case-control study, 157 T2DM patients who were hospitalized in the Department of Endocrinology and Department of Cardiovascular Medicine in Affiliated Hospital of Chengde Medical University from December 2020 to July 2021 were included, including 80 T2DM patients without CHD(T2DM-0 group); 77 T2DM patients with CHD (T2DM-CHD group) were included. Inclusion criteria: (1) The diagnosis of T2DM met the 1999 WHO diabetes diagnostic criteria [15]. (2) The diagnosis of CHD complies with the diagnostic criteria for CHD formulated by the Cardiovascular Branch of the Chinese Medical Association in 2018 [16]. (3) Aged ≥ 18 years. Exclusion criteria: (1) Those with acute infection and stress; those with severe heart and liver failure; those with all degrees of renal insufficiency; those with pregnancy, breastfeeding, and malignant tumors; those who regularly took estrogen, glucocorticoids and other drugs for the past month. (2) T1DM and special types of diabetes. (3) Exclude acute and chronic complications of diabetes.

### Ethical approval and consent to participate

All procedures performed in studies involving human participants were in accordance with the ethical standards of the institutional and/or national research committee and with the 1964 Helsinki declaration and its later amendments or comparable ethical standards. Informed consent was obtained from all individual participants included in the study. The ethical committee of the Affiliated Hospital of Chengde Medical College confrmed the study (the committee’s reference number: CYFYLL2020147).The methods were carried out in accordance with the approved guidelines.

### Information and data Collection

A standardized questionnaire survey was conducted on the research subjects by investigators who received unified training. The data collected included age, sex, course of diabetes, history of hypertension and history of drug. The height, weight and blood pressure were measured by a trained physician with a unified measurement tool and the body mass index (BMI) was calculated, BMI was calculated as the body weight (kg) divided by the square of the height (m2).

Biochemical parameters such as blood urea nitrogen (BUN), serum creatinine (SCr), fasting plasma glucose (FPG), glycosylated hemoglobin A1c (HbA1c), total cholesterol (TC), triglyceride (TG), high-density lipoprotein cholesterol (HDL-C), low-density lipoprotein cholesterol (LDL-C), alanine aminotransferase (ALT) and aspartate aminotransferase (AST) were measured using standard procedures in the hospital clinical laboratory.Triglyceride glucose index ( TyG index) = ln [fasting TG(mg/dL) * FBG (mg/dL)/2], the unit of TG and FPG in the TyG index is mg/dl. All participants had been fasting for 10 h before blood collection. A volume of 5 mL of cubital venous blood was drawn from each patient and transferred to a vacuum blood collection tube.

Determination of serum Asprosin and Nrg-4 levels: The kit for detecting Asprosin serum levels by enzyme-linked immunosorbent assay was purchased from abcam company. The kit for detecting Nrg-4 serum level by ELISA was purchased from Phoenix Company of the United States.

### Statistical methods

SPSS 26.0 (IBM, USA) was performed to analyze data. Continuous variables were presented as mean ± standard deviation (SD) or median (25th and 75th percentiles: P25, P75) in the case of normal or non-normal distribution, and differences between the two groups were examined by independent-sample t-test or Mann–Whitney U test correspondingly. Categorical variables were expressed as counts and percentages, and the comparison between groups was analysed by chi-square test. The correlation between Asprosin or Nrg-4 and other clinical variables was analyzed by Spearman correlation analysis. Binary logistic regression analysis was used to analyze the influencing factors of CHD in hospitalized T2DM patients according to 3 Models (Model 1 was adjusted for gender, age, duration of diabete and history of hypertension. Model 2 was adjusted for gender, age, duration of diabete, history of hypertension, BMI, FPG, HbA1C and TyG index. Model 3 was adjusted for gender, age, duration of diabete, history of hypertension, BMI, FPG, HbA1C, TyG index, TC, TG, HDL-C, LDL-C, ALT, AST, SCr, BUN). The receiver operating characteristic (ROC) curve was used to analyze the predictive value and optimal cut point value of Asprosin and Nrg-4. *p* < 0.05 means the difference is statistically significant.

## Results

### Basic characteristics of the two groups of people

The clinical characteristics and biochemical indicators of the 80 diagnosed T2DM patients and 77 diagnosed T2DM-CHD were showed in Table 1. Patients in T2DM with CHD tended to be older, had diabetes for a longer duration, higher BMI, FPG, TG, TyG index, SCr, BUN levels. These patients were more likely to have a history of hypertension and oral blood pressure, lipid-lowering, and glucose-lowering drugs. Compared with T2DM-0 group, patients with T2DM-CHD had significantly lower in TC and LDL-C. There were no significant differences between the two groups in gender, HbA1C,HDL-C, and insulin application (*p* > 0.05).

**Table 1:** Clinical Characteristics of All Patients with T2DM with and without CHD

Variable	T2DM-0 group(n=80)	T2DM-CHD group(n=77)	*p* value
Male(%)	40(50)	42(54.5)	0.569
Age(year)	55.51±6.36	59.59±7.10	<0.001
Diabetes duration (year)	6(1,11.75)	12(6,16)	<0.001
HA (%)	37(46)	54(70)	0.003
BMI(kg/m^2^)	24.47(23.05,27.24)	26.89(24.58,29.38)	<0.001
FPG (mmol/L)	8.50(7.00,10.65)	10(7.55,12.40)	0.039
HbA1C(%)	9.50(7.95,10.80)	8.80(7.30,10.25)	0.065
TyG index	9.52±0.67	9.80±0.77	0.022
TC (mmol/L)	4.76(3.94,5.96)	4.11(3.12,4.95)	<0.001
TG (mmol/L)	2.11(1.37,2.81)	2.46(1.64,3.29)	0.032
HDL-C (mmol/L)	1.02(0.84,1.20)	0.95(0.80,1.16)	0.12
LDL-C (mmol/L)	2.77(1.96,3.49)	2.04(1.54,2.93)	0.001
ALT (U/L)	21.10(15.06,32.28)	24.52(15,35.50)	0.382
AST (U/L)	21.51(15.80,28.69)	24.67(20.27,32.95)	0.076
SCr (umol/L)	57.40(45.90,65.88)	64.50(51.75,79.55)	0.005
BUN (mmol/L)	5.55(4.57,6.51)	5.79(5,7.66)	0.026
Antihypertensive drugs(%)	28(35)	50(64.90)	<0.001
Statins(%)	7(8.70)	46(57.90)	<0.001
Oral hypoglycemic drugs(%)	64(80)	73(94.81)	0.005
Insulin application(%)	48(60)	43(55.84)	0.598

Data presented as means ± SD, median (P25, P75), or n (%); t-tests for continuous data, Mann–Whitney U tests for abnormally distributed variables, and chi-square test for categorical data. T2DM-0 group: type 2 diabetes mellitus patients without coronary heart disease; T2DM-CHD group: type 2 diabetes mellitus patients with coronary heart disease; HA: Hypertension; BMI: body-mass index; FPG: fasting plasma glucose; HbA1c: glycated hemoglobin A1c; TC: total cholesterol; TG: triglyceride; HDL-C: high-density lipoprotein cholesterol; LDL-C: low–density lipoprotein cholesterol; ALT: alanine aminotransferase; AST: aspartate aminotransferase; BUN: blood urea nitrogen; SCr: serum creatinine. *p* < 0.05 was considered statistically significant

### Serum asprosin and Nrg-4 levels in two groups of people

**Fig. 1:**
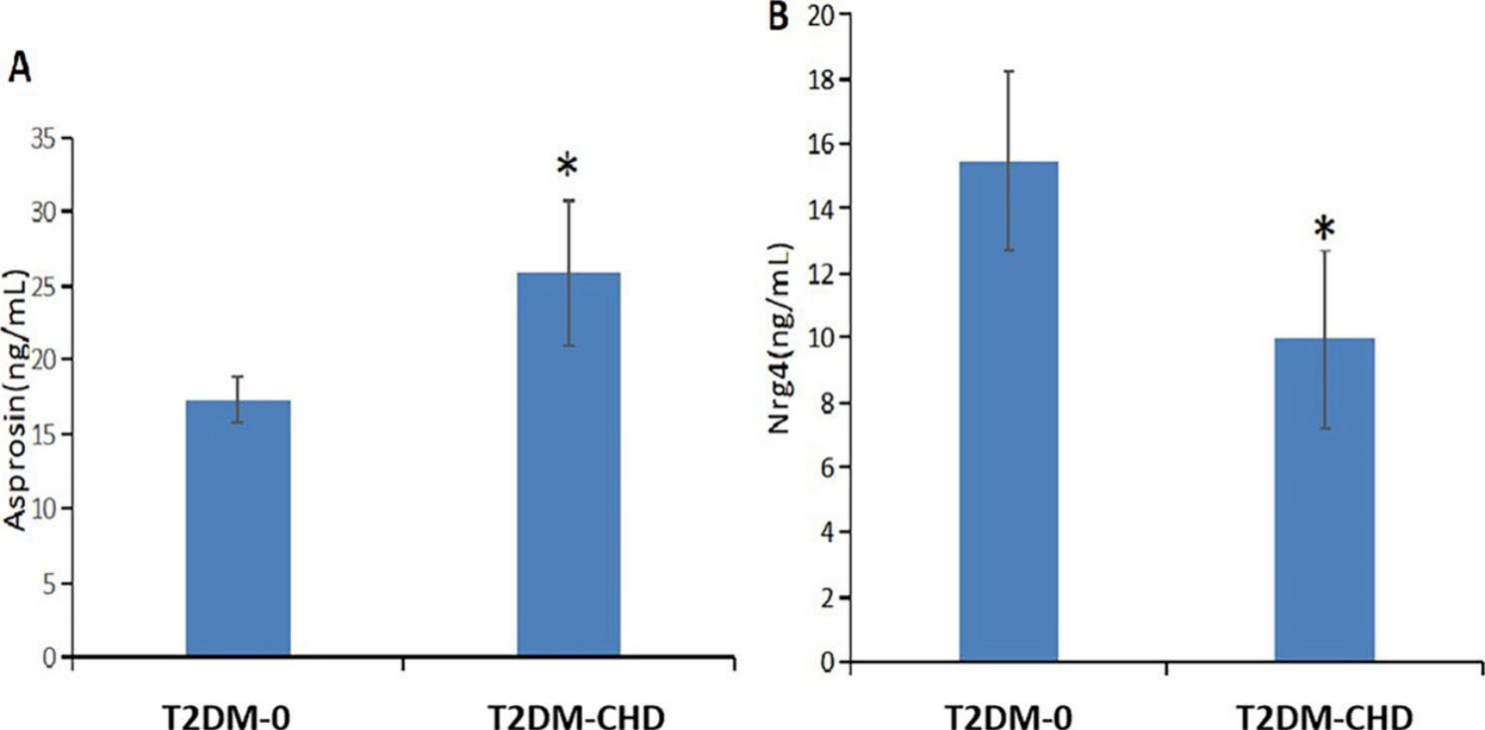
Levels of Asprosin (A) and Nrg-4 (B) in plasma of T2DM-0 group and T2DM-CHD group. *p* value < 0.05 was considered significant

Compared with the T2DM-0 group, the serum Asprosin level in the T2DM-CHD group was significantly increased with 15.86 (13.24, 20.05)ng/ml in T2DM-0 vs. 22.63 (13.94, 27.97) ng/mL in T2DM-CHD, and the difference was statistically significant (p < 0.01).(Fig. 1A) The level of Nrg-4 was significantly decreased in the T2DM-CHD group with 13.38 (11.00, 17.18)ng/ml in T2DM-0 vs. 9.12 (7.55, 12.80) ng/mL in T2DM-CHD, and the difference was statistically significant (p < 0.01). (Fig. 1B)

### Correlations between asprosin or Nrg-4 levels and the clinical basic indicators

According to Spearman correlation analysis, Asprosin levels were positively correlated with the diabetes duration(r = 0.301, *p* < 0.001), history of hypertension(r = 0.204, *p* = 0.01), FPG (r = 0.463, *p* < 0.001), HbA1C (r = 0.24, *p* = 0.03), TG (r = 0.182, *p* = 0.023), TyG index (r = 0.327, *p* < 0.001) and BUN (r = 0.161, *p* = 0.045), and negatively correlated with ALT (r = -0.224, *p* = 0.005). Asprosin levels were not associated with BMI, TC, LDL-C, AST and other variables (Table 2).

**Table 2:** Correlation between Asprosin, Nrg-4, and other variables

variable	Asprosin		Nrg-4	
	r	*p* value	r	*p* value
Male(%)	0.112	0.163	-0.001	0.992
Age(year)	0.141	0.077	-0.074	0.358
Diabetes duration(year)	0.301	<0.001	-0.15	0.06
BMI(kg/m2)	0.142	0.077	-0.18	0.024
HA (%)	0.204	0.01	-0.179	0.025
FPG(mmol/l)	0.463	<0.001	-0.652	<0.001
HbA1C (%)	0.24	0.03	-0.255	0.001
TC (mmol/l)	0.008	0.925	0.038	0.64
TG (mmol/L)	0.182	0.023	-0.355	<0.001
HDL-C (mmol/L)	-0.045	0.578	0.177	0.026
LDL-C (mmol/L)	-0.003	0.971	0.037	0.644
TyG index	0.327	<0.001	-0.534	<0.001
ALT (U/L)	-0.224	0.005	-0.063	0.433
AST (U/L)	-0.042	0.605	-0.13	0.106
SCr (umol/L)	0.144	0.071	0.053	0.512
BUN (mmol/L)	0.161	0.045	0.003	0.975
Oral hypoglycemic drugs(%)	0.051	0.529	-0.012	0.883
Insulin application(%)	0.156	0.051	-0.029	0.722

A Spearman correlation was performed and a value of *p* < 0.05 was considered as statistically significant. HA: Hypertension; BMI: body-mass index; FPG: fasting plasma glucose; HbA1c: glycated hemoglobin A1c; TC: total cholesterol; TG: triglyceride; HDL-C: high-density lipoprotein cholesterol; LDL-C: low–density lipoprotein cholesterol; TyG index: triglyceride glucose index; ALT: alanine aminotransferase; AST: aspartate aminotransferase;BUN: blood urea nitrogen; SCr: serum creatinine.

Spearman correlation analysis showed that Nrg-4 levels were negatively correlated with the history of hypertension(r = -0.179, *p* = 0.025), BMI (r = -0.18, *p* = 0.024), FPG (r = -0.652, *p* < 0.001), HbA1C (r = -0.255, *p* = 0.001), TG (r = -0.355, *p* < 0.001), TyG index (r = -0.534, *p* < 0.001), and positively correlated with HDL-C (r = 0.177, *p* = 0.026). Nrg-4 levels were not associated with TC, LDL-C, ALT, AST and other variables (Table 2). In addition, Asprosin was negatively correlated with Nrg-4 (r=-0.329, *p* < 0.001).

### Logistic regression analysis of the effects of asprosin and Nrg-4 on T2DM-CHD

The association between the levels of Asprosin and Nrg-4 and T2DM-CHD was analyzed in 3 Models. After adjustment for Model 1, a 1 unit (0.1ng/ml) increase in Asprosin significantly increased the rate of existence of T2DM-CHD by 1.072-fold (OR 1.072, 95% CI: 1.023–1.125; *p* = 0.004). Plasma Asprosin concentrations were significantly associated with the development of T2DM-CHD even after controlling for gender, age, duration of diabete, history of hypertension, BMI, FPG, HbA1C, TyG index, TG, HDL-C, LDL-C, ALT, AST, Scr, BUN (OR 1.087, 95% CI: 1.018–1.161, *p* = 0.013), indicating that there was a 1.087-fold increase in the odds of having T2DM-CHD for each 1 ng/ml increase in Asprosin levels (Table 3).

**Table 3:** Logistic regression analysis of associations between T2DM-CHD and Asprosin, Nrg-4

Model	Asprosin		Nrg-4	
	OR(95%CI)	*p*	OR(95%CI)	*p*
1	1.072(1.023,1.125)	0.004	0.74(0.658,0.834)	<0.001
2	1.074(1.013,1.139)	0.017	0.665(0.557,0.795)	<0.001
3	1.087(1.018,1.161)	0.013	0.67(0.557,0.806)	<0.001

Model 1,adjusted for gender, age, duration of diabete and history of hypertension;

Model 2,adjusted for gender, age, duration of diabete, history of hypertension, BMI, FPG, HbA1C and TyG index;

Model 3,adjusted for gender, age, duration of diabete, history of hypertension, BMI, FPG, HbA1C, TyG index, TC, TG, HDL-C, LDL-C, ALT, AST, SCr, BUN

p < 0.05 was considered statistically significant

After adjustment for Model 1, the odds of T2DM-CHD decreased by 26% per 1 unit (0.1ng/ml) increase in serum Nrg-4 level (OR 0.74, 95% CI 0.658–0.834; *p* < 0.001). The OR for T2DM-CHD remained statistically significant even after adjusting for all potential confounders (OR 0.67, 95% CI 0.557–0.806; *p* < 0.001) (Table 3).

### Area under the ROC curve and predictive value of asprosin and Nrg-4

**Fig. 2:**
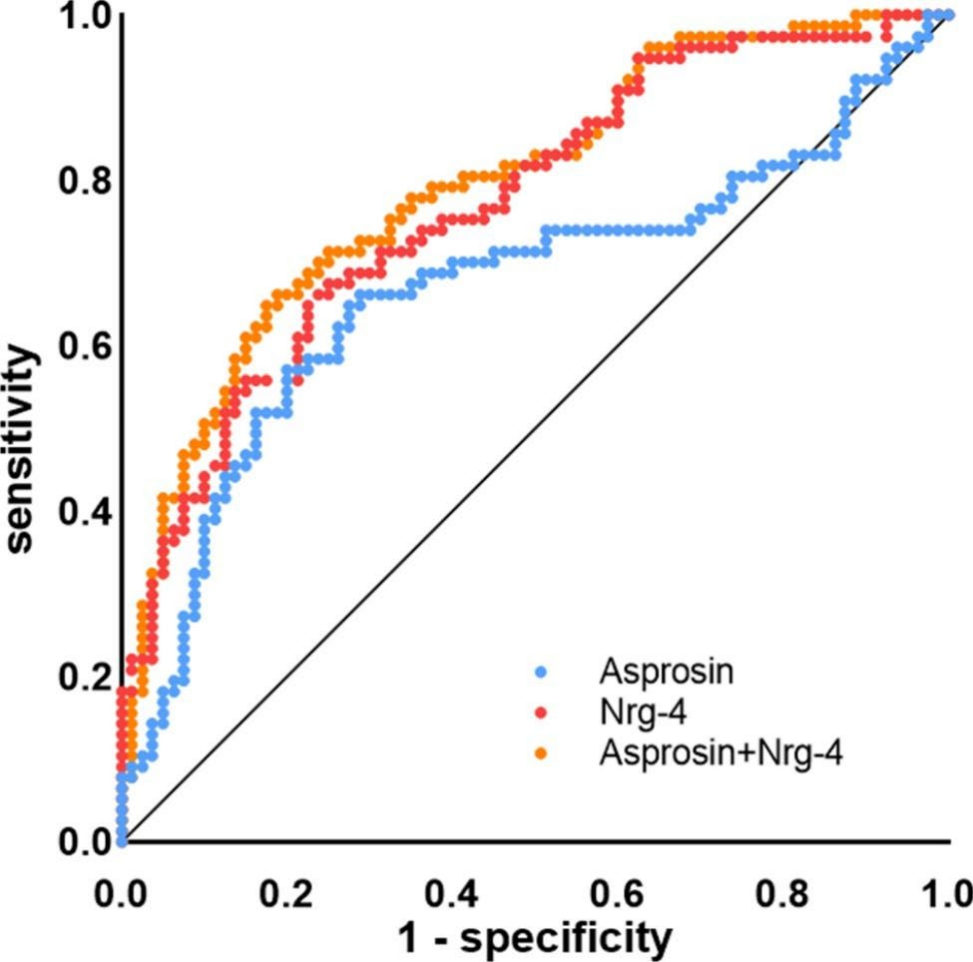
ROC curve analysis of Asprosin, Nrg-4 and their combination in the diagnosis of T2DM complicated with CHD. *p* value < 0.05 was considered significant

To explore the predictive value of circulating Asprosin and Nrg-4 for T2DM-CHD, we analyzed the ROC curves of circulating Asprosin and Nrg-4. The results revealed that the best cutoff value for circulating Asprosin to predict CHD was 19.008ng/ml (sensitivity: 66.2%, specificity: 71.2%, and AUC 0.671), and the best cutoff value for circulating Nrg-4 to predict CHD was 11.175ng/ml (sensitivity: 67.5%, specificity: 75%, and AUC 0.772) in patients with T2DM. The combined prediction was analyzed using a ROC curve. The results revealed that sensitivity, specificity, and AUC of the combination of Asprosin and Nrg-4 for diagnosing T2DM-CHD were 64.9%, 81.2%, and 0.796; such a combination demonstrated significantly improved diagnostic efficacy (Fig. 2).

## Discussion

Adipose tissue secretes a variety of biologically active substances such as adipokines. Previous studies have found that Asprosin, a new type of adipokines secreted by white adipose tissue, is associated with the severity of unstable angina and acute coronary syndrome [17]. This study found that the level of serum Asprosin in the T2DM-CHD group was significantly higher than that in the T2DM-0 group. Although there are few studies at home and abroad on the relationship between Asprosin and diabetes complicated with CHD. However, an Iranian clinical case-control study found that the serum asprosin concentration in the CHD group was significantly higher than that in the healthy group [7]. In addition, compared with the healthy group, a study found that Asprosin was significantly increased in the T2DM-0 group, and it was associated with the ratio of IR and TC/HDL-C (a risk factor for atherosclerosis in CHD) [18]. Therefore, this study provides further evidence of the association between Asprosin and related indicators.

It was found that Asprosin was significantly positively correlated with known cardiovascular risk factors such as diabetes duration, FPG, HbA1C, TG, and TyG index, which was used as a reliable and landmark clinical indicator of IR [19]. Consistent with the results of the Iranian study, serum Asprosin levels in CHD patients were positively correlated with FPG, TG, and HOMA-IR [7]. Basic studies have shown that Asprosin induces β-cell apoptosis by inhibiting β-cell autophagy through adenosine monophosphate-activated protein kinase (AMPK) and mammalian target of rapamycin (mTOR) signaling pathways [20]. Wang et al5 found that Asprosin may lead to β-cell dysfunction and impaired glucose tolerance in patients with T2DM. In this study, it was found that the level of Asprosin was positively correlated with the TyG index, and was significantly increased in the T2DM-CHD group, indicating that IR is not only an important pathological basis of diabetes, but also an important cause of CHD, suggesting that Asprosin may be related to IR, and may play a role in the occurrence of CHD. have important meaning. According to the study by Romere et al., [3] it was found that Asprosin increases the level of circulating glucose by promoting hepatic gluconeogenesis, which provides a basis for the finding that Asprosin is related to FPG and HbA1C in this study. Hypertriglyceridemia is a risk factor for atherosclerosis. This study also found that serum Asprosin levels were significantly positively correlated with TG. However, in the basic data of this population, it was found that the levels of TC and LDL-C in CHD patients were significantly lower, which was contrary to the results of previous studies. It may be related to the significantly increased application of lipid-lowering drugs in this group of patients compared with the T2DM-0 group. A recent cross-sectional study found that there was no correlation between the use of oral hypoglycemic agents, insulin and Asprosin in T2DM patients after multiple linear regression analysis [21], which is consistent with the results of this study. Although another study found that sodium-glucose cotransporter 2 inhibitors could reduce serum asprosin levels in newly diagnosed T2DM patients [22], due to the small number of patients in this study who used sodium-glucose cotransporter 2 inhibitors, no correlation was found, and future studies should increase the sample size.

After adjusting for related confounding factors, Asprosin is still an independent risk factor for T2DM combined with CHD, which indicates that it is closely related to the occurrence of diabetes combined with CAD, which is similar to the results of a domestic study [23]. The study also found the best cut-point value of Asprosin for the diagnosis of diabetes combined with CHD in clinical practice, which has certain value in the prediction and diagnosis of T2DM combined with CHD, but it needs further verification with large samples.

Nrg-4, another novel adipokines, is mainly secreted by brown adipose tissue and is associated with dyslipidemia, IR, inflammation, and oxidative stress, which are involved in the pathogenesis of obesity, diabetes, and metabolic syndrome [24, 25]. The results of the study showed that compared with the T2DM-0 group, the Nrg-4 level in the T2DM-CHD group was significantly lower. Consistent with the results of Tian et al., [26] it was found for the first time that the serum Nrg-4 concentration in CHD patients was significantly reduced, and it was significantly negatively correlated with the SYNTAX score, which reflects the severity of coronary artery disease. In the correlation analysis, this study not only found that Nrg-4 was significantly positively correlated with HDL-C, just as a cross-sectional study found that serum Nrg-4 levels in T2DM patients were positively correlated with HDL-C, [24] but also found that Nrg-4 was positively correlated with HDL-C was significantly negatively correlated with FPG, HbA1C, TyG index, BMI and TG. Previous studies have shown that Nrg-4 has a very significant effect on insulin secretion [27]. Previous studies have also found that high-fat-fed Nrg-4 knockout mice have higher plasma TG concentrations, higher FPG and plasma insulin levels, suggesting that Nrg-4 deficiency can lead to glucose tolerance after diet-induced obesity. Sexual reduction and IR [28]. Conversely, high-fat-fed mice inhibited adipogenesis due to overexpression of Nrg-4, thereby preventing high-fat diet-induced obesity and fatty liver, and improving insulin sensitivity [29]. These studies confirmed that Nrg-4 was closely related to FPG, TG, and TyG index. In addition to animal studies, studies in obese patients have shown that serum Nrg-4 levels are inversely associated with the risk of metabolic syndrome, suggesting that Nrg-4 concentrations may be a protective factor for the development of metabolic syndrome [30]. The above studies further suggest that Nrg-4 may be involved in glucose, lipid metabolism and IR. This study also did not find a correlation between Nrg-4 and oral hypoglycemic drugs and insulin therapy, which is consistent with the previous study not finding that Nrg-4 is related to oral hypoglycemic drugs [31].

Similarly, after adjusting for various confounding factors, Nrg-4 was still an independent protective factor for T2DM complicated with CHD. Tian et al., [26] also found that even if the logistic regression model was adjusted for age, sex, BMI, and HbA1C, the severity of coronary artery lesions in CHD patients was negatively correlated with serum Nrg-4 levels. It is suggested that Nrg-4 may be a protective factor for CAD. This study is consistent with the above findings. Therefore, we speculate that Nrg-4 may be related to the pathogenesis of diabetes and CHD. In addition, the study also found the best cut-point value of Nrg-4 in clinical diagnosis of T2DM complicated with CHD, which has certain value in predicting whether diabetes is complicated with CHD. Studies have shown that Nrg-4 may improve IR and glucose and lipid metabolism through other factors. mechanism to prevent CHD [30]. Correlation analysis showed that Asprosin and Nrg-4 were significantly negatively correlated, and Asprosin combined with Nrg-4 had higher predictive value and higher specificity (81.2%). Although no research has found whether there is a common pathway between the two, whether they are related to the pathogenesis of glucose and lipid metabolism needs further research.

In addition, we found that patients in the T2DM-CHD group were older and had a longer diabetes duration. A previous study found that older age or age at diagnosis and longer diabetes duration proportionally increased the risk of macrovascular events and death, with the greatest risks observed in the oldest age groups with the longest duration of diabetes [32]. However, Logistic regression analysis showed that Asprosin and Nrg4 are still associated with T2DM-CHD after adjusting for factors such as age and diabetes duration. Thus, the level of Asprosin and Nrg4 could be the promising clinical biomarker for predicting T2DM with CHD.

The limitation of this study is a small number of study cases, which limits the conclusions of this study. Furthermore, this was a cross-sectional study, and the effects of the Asprosin and Nrg-4 on the prognosis of T2DM-CHD patients were not observed further. In the future, multicenter, large sample and prospective studies are needed to further confirm the conclusions of this study. In-depth study of its specific molecular mechanism contributes to the early detection of diseases and provides new targets for treatment.

## Conclusion

In conclusion, serum Asprosin level increased and Nrg-4 level decreased in patients with CHD, which is closely related to glucose and lipid metabolism disorder and insulin resistance, and has certain significance for the prediction and diagnosis of T2DM with CHD. Serum Asprosin and Nrg-4 are expected to be potential markers for the diagnosis of T2DM combined with CHD.

## Data Availability

The data used and/or analyzed during the current study are available from the corresponding author on reasonable request.
